# Post-release monitoring: the Brazilian system, its aims and requirements for information

**DOI:** 10.1007/s11248-014-9787-y

**Published:** 2014-03-23

**Authors:** P. P. Andrade, M. A. Melo, E. A. Kido

**Affiliations:** 1 Universidade Federal de Pernambuco, Recife, PE Brazil; 2 Universidade Federal de Campina Grande, Patos, PB Brazil

**Keywords:** GMO, Genetically modified organism, Commercial release, Cost/benefit

## Abstract

The Brazilian National Biosafety Committee approved in 2011 a new post release monitoring system for environmental releases of genetically modified organisms. It has a number of novel features in comparison with other established or proposed systems. The new system also allows the proponent to ask for monitoring exemption. General surveillance forms the basis of the monitoring system, similar to the European model, but differs markedly in the way it operates. While the European proposal is based on monitoring measurable variables extracted from environmental observations, from baselines previously established for multiple protection targets, the Brazilian system uses direct alerts of damage, without the aid of baseline values. The strength of the Brazilian form of monitoring is the possibility of generating an information network with the effective participation of many actors from the monitored area. A network constituted by highly qualified members, as proposed elsewhere, is too complex and unrealistic in Brazil and in many other countries. In conclusion, the Brazilian monitoring system is flexible and can be adjusted to the Brazilian reality over the next years, as a response to the ever growing experience in monitoring. It also meets the demands of the Brazilian society for transparency, rational use of resources, opportunity for national companies, and food and environmental biosafety.

Genetically modified organisms (GMOs) are strictly regulated in most countries and the risks for the environment and health are extensively evaluated. To date, harm attributable to a GMO has not been substantiated for any commercial release, either to the environment or to health. Effectively, hundreds of different GM plants are now in the market and all of them have been assessed as having negligible risks (see CERA Database, available at http://www.cera-gmc.org/?action=gm_crop_database) and as safe as their non GM counterparts. Nevertheless, long term effects and other factors are relevant to GMO regulatory decision-making (Raybould 2012). Consequently, both Europe (EFSA 2011) and Brazil have adopted monitoring as an integral step in risk management. In the case of Brazil, both public opinion and the requirement of Article 16 of the Cartagena Protocol on Biosafety to the Convention on Biological Diversity (Cartagena Protocol) for post release monitoring (CBD 2000) were used as the basis of the decision to adopt this strategy.

GMO monitoring is generally regarded as an opportunity to identify new unanticipated risks derived from the interaction of a given GMO with a complex environment over extended areas, by detecting unforeseeable harmful effects on the environment and human health. (FAO 2011; Wilhelm et al. 2003). Moreover, it can also be used to evaluate performance, what is usually called product stewardship. On 2011 the Brazilian National Biosafety Committee (CTNBio) approved a novel system for post release GMO monitoring (available at http://www.ctnbio.gov.br/index.php/content/view/18000.html). While still following the general European approach to the question, it differs in a number of important aspects compared with the European model and other established or proposed systems. The Brazilian system adopts general surveillance (GS) as the core process in GMO monitoring, taken into account that most GMOs have been considered up to date to be as safe as their non-GM counterpart. Since all risks have been considered negligible for approved commercial releases, CTNBio decided that no case-specific monitoring (CSM) was required. This approach is similar to those adopted by the European Union and proposed elsewhere (FAO, *op.cit.*).

The Brazilian system is novel in does not require establishment of baselines of measurable variables extracted from environmental observations as parameters for the detection of harm. Instead, it uses actual damage as an indicator of possible harms derived from the GMO. This was only possible due to the realization by CTNBio, based on more than 10 years of intensive use of GM crops in Brazil and elsewhere, that few uncertainties remain for consideration by risk managers. On the other hand, baseline establishment and follow up were considered to be unrealistic due to costs and to the specific dynamics of modern agriculture. In addition, there are extensive areas under cultivation with GM crops of many types. Brazil has currently more than 40.3 mi hectares of GM crops (Celeres 2013), and more than 20 events of GM maize, cotton and soybean (see http://www.ctnbio.gov.br/index.php/content/view/12492.html).

As in the European proposal, the CTNBio regulation suggests as appropriate the following sources of information for GS:

- I—reports on specific technical meetings held to assess the technology employed by users;
- II—use of accessible and appropriate communication media or consumer service made available by the applicant;
- III—questionnaires to the technology users and other actors involved in the process, prepared by the applicant;
- IV—report containing summary and references to scientific literature published about the GMO event, or related events in peer reviews, or government agencies reports;
- V—official notification systems; and
- VI—other monitoring tools in line with the GMO use application.

Item III is similar to the farmers questionnaire, a subject of great interest in Europe and with which EFSA was involved since 2007, to help define the parameters for GS (Waigmann et al. 2012). The other parameters are broadly equivalent to those proposed by different sources.

Flowchart in Fig. 1 summarizes the steps leading to the adoption of a monitoring plan for a given GMO coming to the market or to its exemption, as well as the various decisions and actions within the plan.

**Fig. 1:**
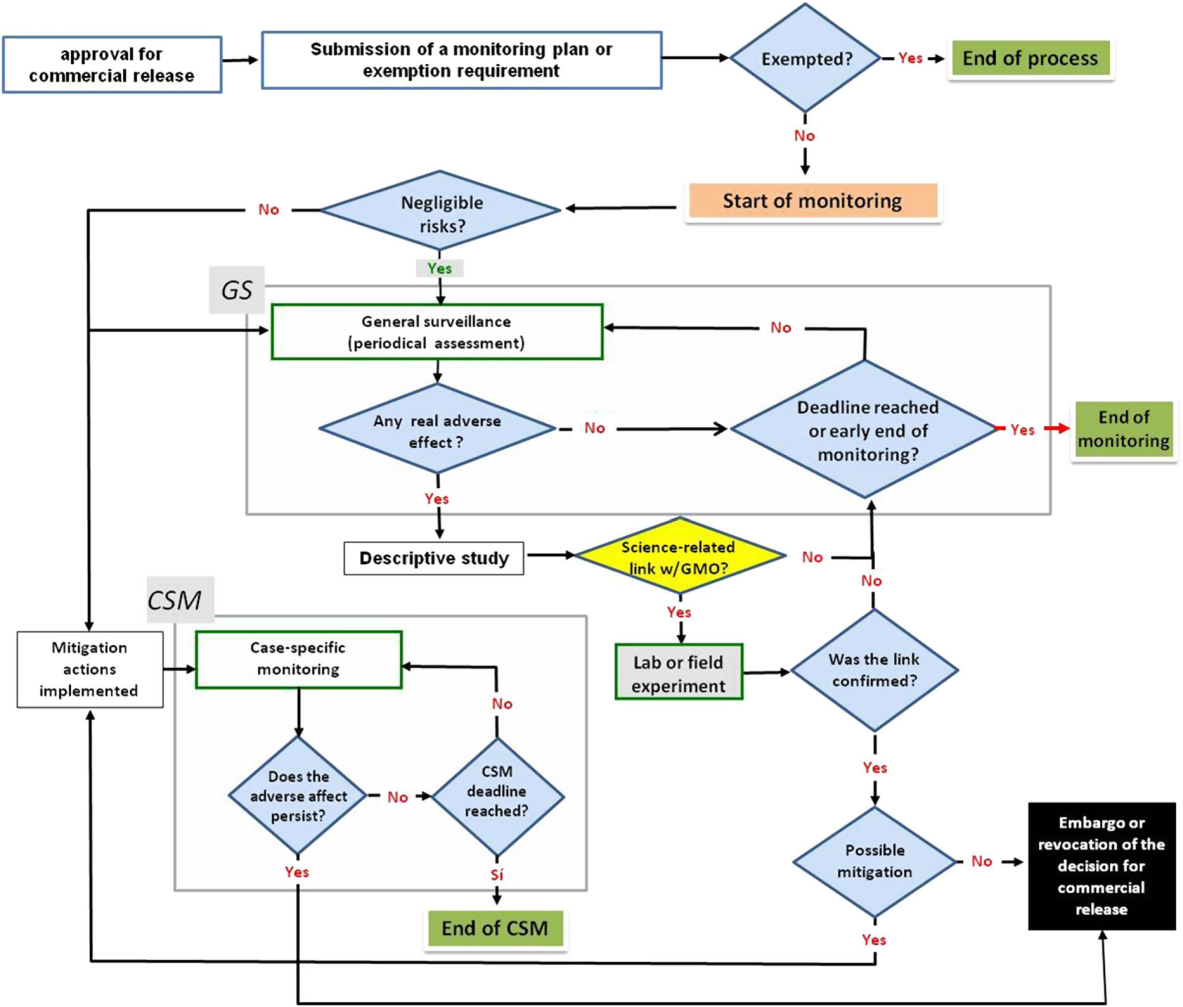
Actions and decision steps in the approval and implementation of a post release monitoring plan, or to its exemption, as in the Brazilian CTNBio Regulation #9. The monitoring plan must be submitted to CTNBio only when the risk assessment has been completed and the authorization of commercial release has been granted. If the exemption is granted, the process ends. Otherwise, the monitoring plan usually starts as a GS and will continue until the deadline is reached, unless damages associated with GMOs are observed during the period; in this case a CSM plan is enabled. Other decision steps and actions are depicted in the figure

The Brazilian post release monitoring system also allows in certain cases exemption from monitoring. There are already some cases in which exemption could be justified, for example, GMOs that are not released alive into the environment (e.g., microorganisms grown in fermentation tanks), or for those that do not proliferate in the environment (such as transgenic mosquitoes that die after a few days and whose progeny also die). A recent case of exemption relates to a GM yeast that was withdrawn from the market. This will certainly be the case of many GM plant events that will be substituted by more modern varieties, having single or stacked events. Over time, other cases of exemption will have to be considered by CTNBio. Nevertheless, a transgenic mosquito is currently been evaluated by CTNBio and its proponent also decided to develop a monitoring plan, although exemption may have been considered.

It is important to keep in mind that the Brazilian law does not require GMO post release monitoring. Both the Brazilian and the international experience on the cultivation and use of various GMOs continue to build and will certainly lay down a solid foundation of risk management. This will determine whether it is productive to monitor a new GM, or if the information available will allow the exemption of this procedure. The exemption option is thus a useful feature as it is aligned with the best science, fits the previous risk assessment and avoids the expenditure of time, resources and personnel on monitoring products with a history of safe use, or which cannot be monitored by technical issues. Mandatory monitoring may add to the regulatory burden without providing additional protection to humans, livestock or the environment.

Case-specific monitoring may be triggered by the occurrence of damage with a proven link to the GMO. According to the Brazilian proposal, early damage warnings generated by the monitoring network must be first assessed by the company (which conducts monitoring and pays its cost) and a technical report must then be submitted to CTNBio for each alert. If a scientific plausible link between the damage and the monitored GMO is found, laboratory experiments are conducted to test the hypothesis (with oversight by CTNBio). If there is corroboration of causality, mitigation measures should be adopted and the CSM is therefore triggered. Alternatively, CSM can be triggered from the very start of the monitoring plan if non-negligible risks are identified in the risk assessment prior to commercial release. The pertinent decisions and actions underlying GS and CSM are summarized in Fig. 2 below (part of the flowchart in Fig. 1).

**Fig. 2:**
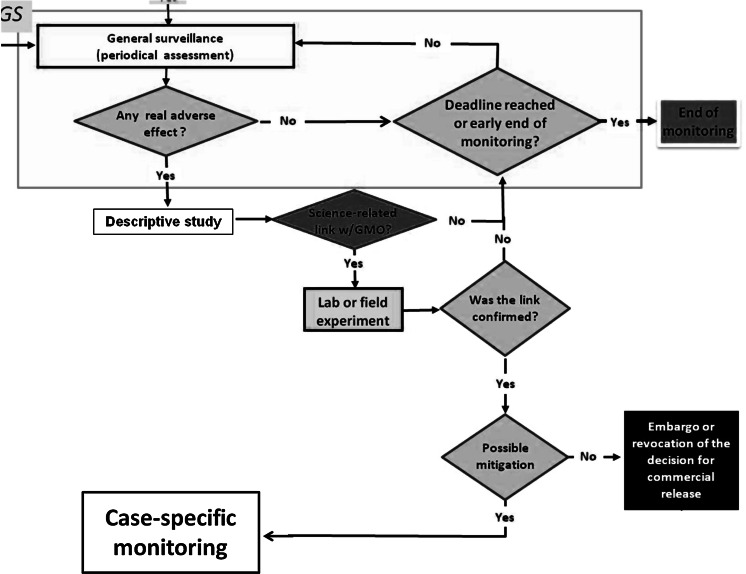
Excerpt taken from the general flow chart showing how a damage alert (real adverse effect) generated during GS can trigger experiments and ultimately CSM. An alert requires the finding of an adverse effect (damage); if it is substantiated, the alert generates a technical report that CTNBio, together with the company, must analyse to find an eventual causation link between the GMO and the harm. If there is scientific basis for causation, specific experiments should be performed, whose results will determine the subsequent actions

Deadlines are also an important cause of concern. For example, monitoring of a yeast grown in industrial tanks may need to be no greater than 2 years. Maize, soybean and other annual crops could be monitored for 5 years, but since farmers usually rotate crops and change events, it is generally difficult to monitor the same area for a large period. To establish deadlines for perennial trees is a real challenge, as well as for sugar cane. Another controversial issue is the substitution of single events by their higher order stacked product in monitoring plans, a strategy allowed in the Normative. CTNBio hopes that a fresh flow of information derived from the monitoring plans now under its supervision will shed more light on these issues.

In conclusion, the strength of the Brazilian monitoring system is the possibility of generating an information network with the effective participation of many stakeholders from the monitored areas. Although the European proposal also describes a network, it is much more restricted because it is responsible for creating baselines and is strongly dependent on high qualification of its members. To build such a network is complex and unrealistic in Brazil and in many other countries, especially among government agencies responsible for technical training on agricultural practices, seed sellers, producers’ associations and other forms of social organization. The quality of the network will be evaluated by the consistency of the alerts. On the other hand, the network is not open to the public, as it has to be approved by CTNBio. Thus, a well-built network represents an invaluable source of information, without the need to establish expensive baselines and with minimizing the number of false damage reports. The Brazilian post-release monitoring system seems therefore able to be implemented as a rigorous, thorough and effective protocol, in spite of only triggering significant action if case of substantiated damage. It is important to keep in mind that possible damages will be restricted to the areas where the specific event under monitoring was adopted in large scale and that no such damages where ever reported for previous commercially released GMOs.

Irrespective of how complex the post release monitoring could be, it will always be limited in its ability to cover all possible unanticipated risks. Above all, it will not be able to eliminate all uncertainty (Sanvido et al. 2011), but which are presently minimal. Therefore, it is expected that exemption will be increasingly asked for by proponents of new products, except for stewardship (which can also signal unanticipated risks).

The new monitoring system will have costs certainly compatible with its possible results. It is also flexible and can be adjusted to the Brazilian reality over the next years, as a response to the ever growing Brazilian and international experience in monitoring. It also meets the demands of the Brazilian society for transparency, rational use of resources, opportunity for national companies and food and environmental biosafety, and without using an excessive regulatory burden.
